# Photoactive Nanomaterials Inspired by Nature: LTL Zeolite Doped with Laser Dyes as Artificial Light Harvesting Systems

**DOI:** 10.3390/ma10050495

**Published:** 2017-05-04

**Authors:** Leire Gartzia-Rivero, Jorge Bañuelos, Iñigo López-Arbeloa

**Affiliations:** Molecular Spectroscopy Laboratory, Department of Physical Chemistry, University of the Basque Country UPV/EHU, 48080 Bilbao, Spain; leire.gartzia@ehu.es (L.G.-R.); inigo.lopezarbeloa@ehu.es (I.L.-A.)

**Keywords:** hybrid nanomaterials, luminescent antennae, energy transfer, LTL zeolite, laser dyes, stopcock molecule

## Abstract

The herein reported work describes the development of hierarchically-organized fluorescent nanomaterials inspired by plant antenna systems. These hybrid materials are based on nanostructured zeolitic materials (LTL zeolite) doped with laser dyes, which implies a synergism between organic and inorganic moieties. The non-interconnected channeled structure and pore dimensions (7.1 Å) of the inorganic host are ideal to order and align the allocated fluorophores inside, inferring also high thermal and chemical stability. These artificial antennae harvest a broad range of chromatic radiation and convert it into predominant red-edge or alternatively white-light emission, just choosing the right dye combination and concentration ratio to modulate the efficiency of the ongoing energy transfer hops. A further degree of organization can be achieved by functionalizing the channel entrances of LTL zeolite with specific tailor-made (stopcock) molecules via a covalent linkage. These molecules plug the channels to avoid the leakage of the guest molecules absorbed inside, as well as connect the inner space of the zeolite with the outside thanks to energy transfer processes, making the coupling of the material with external devices easier.

## 1. Introduction

The development of photoactive nanomaterials capable of mimicking the functions and mechanisms present in nature has become a major challenge for scientists of different fields [1,2,3]. These systems show an incredibly high efficiency and perfection, which tempted the scientific community to simulate them by designing artificial systems that can further guarantee environmentally-friendly technological progress. Photosynthetic organisms present in plants, for instance, appear as the most sophisticated solar energy storage systems in nature due to their unique ability to harvest solar radiation and transform it into chemical energy. The light is absorbed by antenna systems composed of a few hundred chlorophyll molecules embedded in a protein environment, which keeps the photoactive moieties well arranged and with the proper orientation to transfer the excitation energy efficiently to a specific reaction center [4,5]. Nowadays, many efforts are being made to fully understand the complex process of photosynthesis, since it involves many other fundamental processes necessary for the proper performance of the complete system [6]. In this regard and taking inspiration from plant antenna systems, many imaginative attempts have been tested to build artificial luminescent antennae, among them self-assembled nanofibers [7], dendrimers [8], luminescent metal complexes [9], DNA nano-scaffolds [10], dyes embedded in polymer matrices [11] and host-guest hybrid systems [12].

**Figure 1:**
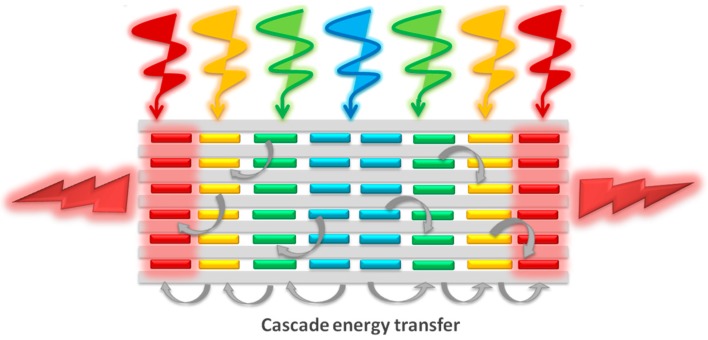
Schematic overview of an artificial luminescent antenna.

One of the main requirements for the proper functioning of an artificial antenna system is the ability to harvest and transport the light to an acceptor moiety with the desired energy (Figure 1). In this sense, the excitation energy transfer (EET) appears as the key factor ruling the effectiveness of the process. Therefore, a thorough understanding of the parameters controlling this phenomenon is necessary, such as the ratio of donors and acceptors, interchromophoric distances, relative orientations and spectral overlap [13]. The accurate control of all of these variables leads to the development of competitive and efficient antenna systems with potential applications in photonics, optical coding, biosensing, catalysis, photosynthetic, logic devices and theranostics, among others [14,15,16].

As an attempt to develop competitive artificial antenna systems, the design of photoactive organic-inorganic hybrid materials shows promising possibilities to mimic the perfection and effectiveness of the antenna systems present in plants. In the herein presented work, the protein matrix of the natural systems has been replaced by a zeolite host (Linde Type L, LTL) of nanometric dimensions, which protects the dyes and provides a significant degree of arrangement [17,18]. Moreover, with respect to the photoactive moiety, responsible for interacting with the light, the chlorophyll molecules have been replaced by fluorescent molecules. Indeed, and thanks to the advances in organic chemistry, nowadays, there is a wide chart of commercially-available laser dye families, which cover the ultraviolet, visible and near-infrared regions of the electromagnetic spectrum [19].

The incorporation of an organic photoactive guest into a nanostructured solid matrix is an active area of research, reflected by the high number of related publications [20,21,22]. The strategy of encapsulation can avoid the formation of aggregates, improve the chemical and thermal stability of the dye and force it to adopt a preferred orientation, creating highly organized photoactive hybrid materials. Furthermore, the synergism between the organic and inorganic components could be used to monitor the physicochemical properties of the solid framework taking advantage of the sensing capabilities of the fluorophore [23].

**Figure 2:**
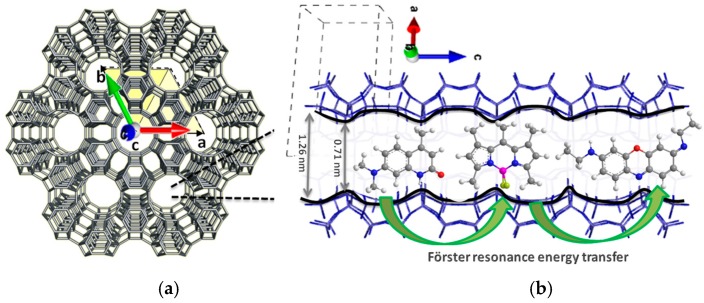
Top view of the LTL zeolite framework, illustrating the hexagonal structure (**a**); and the side view of the channel with a van der Waals opening of 0.71 nm at the smallest and 1.26 nm at the widest place (**b**), suitable for allocating luminescent fluorophores.

Among the natural and synthetic zeolites discovered up to date, the LTL zeolite appears as a suitable candidate to allocate fluorescent dyes. Its unidimensional channel system running along the main axis of the crystal and a cylindrical pore size of a diameter of around 7.1 Å provide a constrained framework in which the molecules fit like a glove, thereby rendering well-organized materials (Figure 2). Therefore, this kind of molecular sieve is an excellent candidate for shape-selective hosting guests (such as ions, metals or organic molecules). The LTL zeolite crystals are usually achieved by hydrothermal synthesis (even assisted by microwave heating) with a tunable size and morphology [24,25,26,27,28].

In this work, we aim to build hybrid photoactive materials as artificial antennae by the sequential insertion of luminescent dyes, working in the blue (oxazole and carbostyril), green-yellow (BODIPYs) and red-edges (oxazines and modified BODIPY) of the electromagnetic spectrum, in the unidimensional channels of LTL zeolite synthesized by microwave heating with a tunable size and morphology. This strategy allows harvesting light over a broad spectral range and transferring efficiently via the Förster resonance energy transfer (FRET) mechanism in a cascade-like unidimensional process from the center to the crystal ends (Figure 1 and Figure 2). The encapsulation of the dyes ensures short donor-acceptor distances to switch on such FRET. An accurate control of the energy transfer efficiency (by means of the donor-acceptor spectral overlap and ratio), as well as an adequate selection of the combined dyes (mainly the final acceptor red-emitting dye on the basis of its fluorescence efficiency) allows a fine modulation of the emission window, from predominant red emission to white-light, and opens the door to a new generation of photoactive materials capable of modulating the emission light output. Moreover, a further degree of organization is provided by the covalent linkage of a stopcock molecule working in the red-edge of the electromagnetic spectrum, which acts as the final energy acceptor and allows the connection of the material to external devices. Therefore, the resulting dye-doped LTL zeolite materials are highly versatile and potential candidates to be used as tailor-made devices for multiple applications. 

## 2. Results and Discussion

### 2.1. Energy Transfer across the Channels of Dye-Doped LTL Zeolite

**Figure 3:**
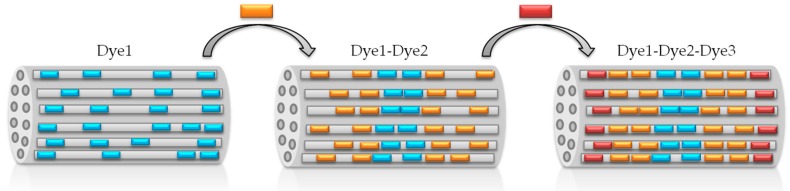
Sketch of the sequential insertion of the fluorophores into the LTL zeolite channels.

The first step to develop antenna systems and white-light-emitting devices is to select dyes working in different regions of the electromagnetic spectrum and susceptible -of promoting efficient intermolecular energy-transfer processes within the LTL zeolite channels [29,30,31]. The energy transfer takes place via dipole-dipole coupling, better known as the through-space Förster mechanism, where the main requirement is the proper spectral overlap between the emission spectrum of the donor and the absorption spectrum of the acceptor (see Figures S1–S3 in the Supporting Information showing the spectral overlaps of the chosen dye combinations) [13]. Moreover the selected dyes should be small enough to diffuse along the channels, but large enough to avoid the formation of aggregates (mainly the non-fluorescent H-type). To ensure broadband absorption along the whole UV-Vis spectral region, three different dyes were successively allocated in the LTL zeolite pores. As energy donors, oxazole (Dmpopop) and carbostyril (C165) dyes have been considered in the UV-blue region, whereas in the green-yellow part, BODIPYs (PM546 and PM567) have been selected as first acceptors and ulterior energy donors. Lastly, as final energy acceptors, red-emitting oxazines (Ox1 and Ox4) were chosen. Table 1 summarizes the donor-acceptor dye loading and ratio, as well as key parameters to describe the FRET probability (such as spectral overlap or critical transfer distance) between the donor-acceptor pairs in each of the herein-tested antennae. The sequential insertion of the fluorophores leads to a hierarchically-organized material in which the UV-blue donors are placed in the center of the crystals, flanked by the green-yellow dyes, and with the red acceptors localized at the edges of the LTL zeolite crystal (Figure 3). In fact, the dimension and constrained environment of the channel framework not only avoid the dyes sliding one over each other, but also provide a short donor-acceptor distance, which improves the FRET efficiency. Therefore, by properly choosing the dyes to be allocated and playing with the spectral overlap and donor/acceptor ratios of the combined dyes, the FRET efficiency can be finely tuned, and hence, the output emission light of the photoactive material may be modulated accordingly.

#### 2.1.1. Dmpopop, PM567,Ox1-Doped LTL Zeolite

**Figure 4:**
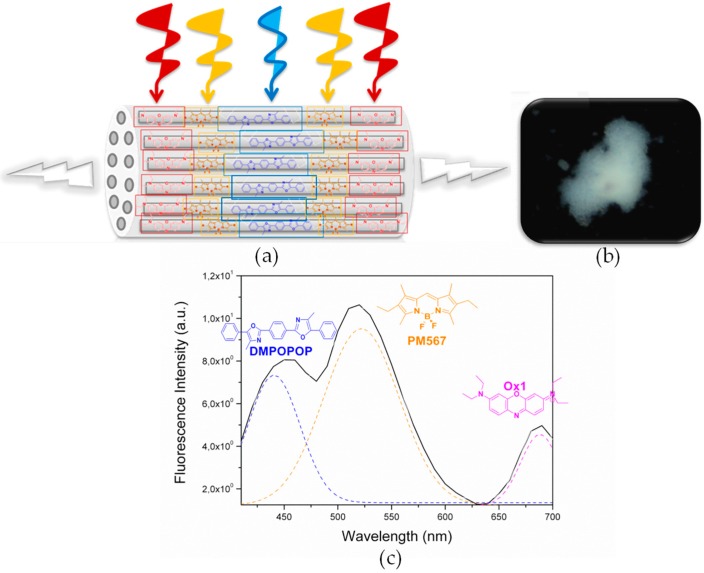
(**a**) Schematic representation of the antenna material prepared by the sequential insertion of three different dyes into disc-shaped LTL zeolite channels; (**b**) Fluorescence image of an agglomerate of the corresponding doped crystals under 350/50-nm excitation and recording the emission with a 400-nm long-pass filter; (**c**) Fluorescence spectrum recorded upon selective excitation of the first donor at 375 nm (dashed lines show the corresponding emission of each dye separately).

The herein presented antenna system is composed of the sequential insertion of Dmpopop-PM567-Ox1 dyes into the zeolitic channels of disc-shaped crystals (see Section 3.2 for details and Table 1) [32]; in particular, following the aforementioned sequential order, first the donor Dmpopop, then the BODIPY and, finally, the oxazine. These dyes kept their identity inside the channels, in spite of the imposed proximity by the constrained framework, ruling out intermolecular aggregation and thereby displaying their characteristic absorption bands spread along the UV-Vis region [32]. As a result, this antenna entails an effective light harvesting over a broad spectral region (Table 1). After selective excitation at the UV region, where just the first energy donor (Dmpopop) absorbs, its own emission is recorded but followed by that from PM567 and the last energy acceptor Ox1 emission band (Figure 4). Therefore, this hybrid material undergoes FRET (see spectral overlap in Figure S1, which enables through-space FRET) as red emission is attained upon excitation far away from the long-wavelength absorption of oxazines, but partial, since the emission from the donors is not completely quenched and the corresponding fluorescence of each chromophore is simultaneously detected. Nonetheless, the sum of all of these emissions covering the whole visible spectrum leads to white-light emission (Figure 4) in which the energy output can be finely tuned over a broad region just using suitable filters [33,34].

#### 2.1.2. C165,PM546,Ox4-Doped LTL Zeolite

In order to transform most of the harvested light into red emission, we decided to improve the energy transfer efficiency by choosing dyes with stronger spectral overlap (see Figure S2 in the Supporting Information and Table 1) and, hence, boost the FRET pathway. For that purpose, we replaced the oxazole by a carbostyril (C165) as the blue emitting energy donor, PM567 by another BODIPY (PM546) in the green-yellow region and Ox1 by another oxazine (Ox4) with improved fluorescence capability as the final acceptor. Indeed, as a consequence of the higher spectral overlap, the critical transfer distance (also known as the Förster distance [13]) increases for each donor-acceptor pair of this antenna with regard to the aforementioned one in Section 2.1.1, anticipating an improved FRET efficiency for the new designed antenna (Table 1). For a proper comparison with the above tested antenna, the rest of the parameters were not altered, which means, the same dye loadings into disc-shaped LTL zeolites (10% of the available free adsorption sites occupied for each dye; see Section 3.5 for details), thus keeping also the same donor/acceptor ratio at 1:1:1. 

**Figure 5:**
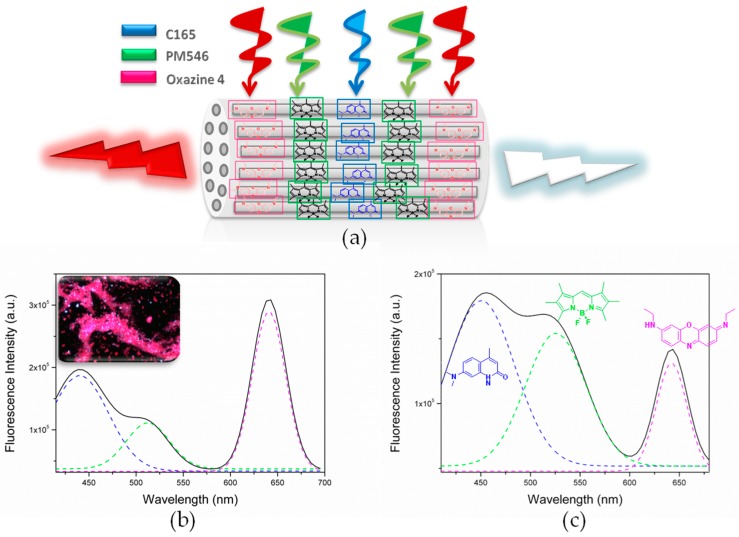
(**a**) Schematic representation of the antenna material prepared by the sequential insertion of three different dyes into disc-shaped LTL zeolite channels. Fluorescence spectrum of (**b**) C165(10%)-PM546(10%)-Ox4(10%)- and (**c**) C165(10%)-PM546(10%)-Ox4(2%)-doped LTL zeolites recorded upon selective excitation of the first donor at 375 nm (dashed lines account for the emission of each free dye). Inset in (**b**): fluorescence image of agglomerated dye-doped zeolite crystals under 350/50-nm excitation and recording the emission with a long-pass filter of 400 nm.

Again, the presence of each dye is verified by the detection of their characteristic bands in the corresponding spectral region spanning almost the whole UV-Vis spectrum (Table 1). After UV excitation, the emission of each chromophore is recorded as a consequence of the expected cascade-like FRET processes from the carbostyril to the neighboring BODIPY and from there to the adjacent oxazine through the unidimensional channel preferably (Figure 5b). However, the ratio between the relative fluorescence intensities of each band changes. Whereas in the previous antenna, the blue and green emission prevails, the red one being less intense, in this case, a much more prominent red-edge emission appears, by far being the most intense peak in the spectrum.

Such evolution is a consequence not only of the ameliorated FRET efficiency, but also of the enhanced fluorescence quantum yield of the final energy acceptor Ox4 with regard to Ox1 [35]. As a result of this synergistic effect, the donor fluorescence quenching by FRET is more effective in profit of the emission from the final and brighter red-edge emitting Ox4 acceptor. Therefore, the control of the energy transfer efficiency together with the improvement of the fluorescence performance of the emitting final energy trap enables the modulation of the emission window, thus passing from the detection of emission bands with similar intensity across the visible region (white-light in Section 2.1.1) to a predominant red emission, keeping always a broadband absorption of the incoming light. Such fine modulation is achieved just by a careful selection of the encapsulated dyes.

Another alternative strategy to modulate the FRET efficiency for a given dye mixture, and hence trigger the output emission from the hybrid material, is changing the donor/acceptor ratio [21], which rules the donor-acceptor distance and, hence, modulates the FRET rate constant [13]. Accordingly, we tested such an approach in the above antenna comprising C165-PM546-Ox4 by reducing the final acceptor loading from 10% to 2% (thereby from a 1:1:1 ratio to 1:1:0.2; see Table 1). Such a decrease of the acceptor amount implies a reduction of the intensity of the red-edge emission from the oxazine upon selective excitation of the first donor Dmpopop (Figure 5c). As a result of this partial energy transfer three emission bands, corresponding from each dye allocated in the zeolitic pores, with similar intensity are recorded simultaneously along the whole visible region, rendering again white-light emission output (as in Section 2.1.1, where a different dye mixture was used). These spectral features are a consequence of a decrease in the energy transfer efficiency, inherent to the diminution of the number of available energy traps responsible for absorbing the excitation energy coming from the donors [36]. Therefore, the emission window can be modulated in a straightforward way from predominant red-edge to white-light emission as in Section 2.1.1, but in this case, adjusting the ratio between donors and acceptors for a given donor-acceptor combination. 

### 2.2. External Functionalization of LTL Zeolite

**Figure 6:**
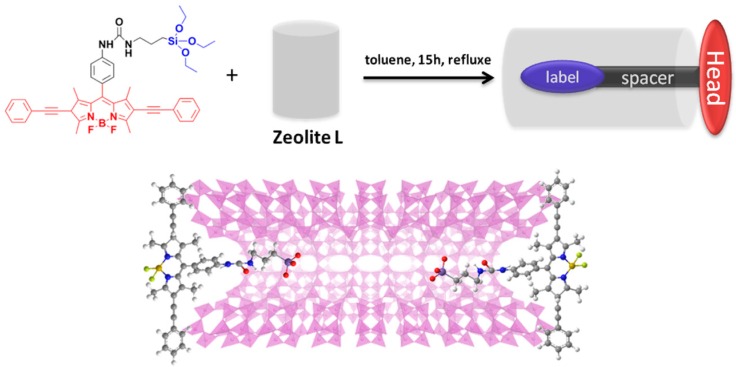
Molecular structure of the stopcock and the reaction principle to attach it to the LTL zeolite channel entrances. The spacer and label are located inside the channel, whereas the head is too big to enter.

Once having checked that the dye occlusion in the LTL zeolite pores is a suitable strategy to develop artificial antennae owing to the promoted FRET pathway, we aim to add a further degree of organization by functionalizing the channel entrances of LTL zeolite with specific tailor-made molecules (stopcock) via covalent linkage (Figure 6) [37,38]. Such molecules grafted at the pores plug the channels to avoid the leakage of the guest molecules adsorbed inside the pores and connect the inner space of the zeolite with the outside thanks to FRET processes, in which the stopcock molecule would act as the final acceptor, making the coupling of the material with other external devices easier. The selective functionalization of the channel entrances is feasible owing to the dissimilar chemical and physical properties of the base of the cylindrically-shaped LTL crystals, bearing the channel entrances, and those of the coat. 

**Figure 7:**
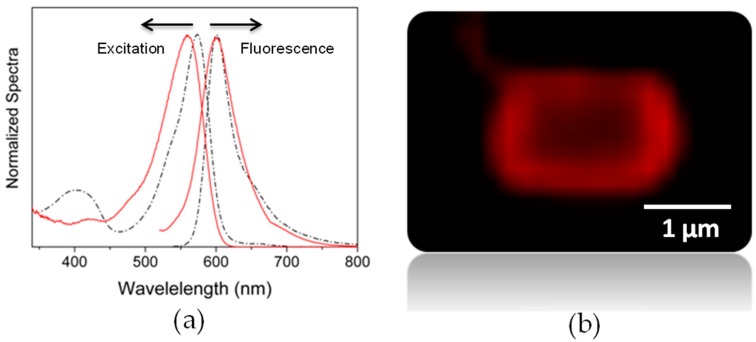
(**a**) Normalized excitation and emission spectra of the BODIPY stopcock grafted to disc-shaped LTL zeolite crystals (solid) and free in toluene (dash dotted); (**b**) Fluorescence microscopy image of a single microsized LTL crystal functionalized with the BODIPY stopcock.

A rational design of these stopcock molecules plays a key role. First it should be able to diffuse into the pores and subsequently covalently graft at the channel entrances by reaction with free silanol (Si–OH) groups, while keeping intact the photophysical properties of the chromophore. At the same time, the emission should be modulated by appropriate functionalization, so as to shift it to the red-edge of the visible to act as suitable energy acceptor of the light harvested inside the zeolite channels [39]. Keeping all of these requirements in mind, we have designed a molecule featuring a label, a spacer and a head moiety, taking as the scaffold the BODIPY dye owing to the exceptional chemical versatility of its dipyrrin core amenable to a wide assortment of synthetic reactions (Figure 6) [40,41]. Accordingly, the head consists in the tetramethylated dipyrrin bearing two acetylenephenyl arms at β-pyrrolic positions to extend the chromophoric π-system and push the spectral bands towards the red-edge. The size of this head is high enough as to avoid its diffusion into the channels and plug the entrances. The label is an organosilane moiety linked to the above head through a spacer bearing a long enough polymethylene chain at the *para* position of a sterically-hindered phenyl grafted at the chromophoric *meso* position (Figure 6). Such a constrained structure around the key *meso* position is essential to avoid any non-radiative pathway associated with the conformational freedom of the aryl and to keep the excellent fluorescence response of BODIPYs [42]. The triethoxysilane label penetrates into the channels and reacts with the zeolitic silanols after hydrolysis and polycondensation reactions, plugging the channel entrances. In fact, the fluorescence microscopy image recorded with microsized crystals (around 2 µm in Figure 7b) reveals that the stopcock molecules are successfully grafted to the microsized LTL zeolite crystal, and this takes place preferentially in the channels’ entrances because the density of the silanol groups is higher than in the coat. Furthermore, the photophysical properties of the stopcock are unaltered after the covalent binding, as its spectroscopic signatures remain similar to those recorded in solution (Figure 7a). 

**Figure 8:**
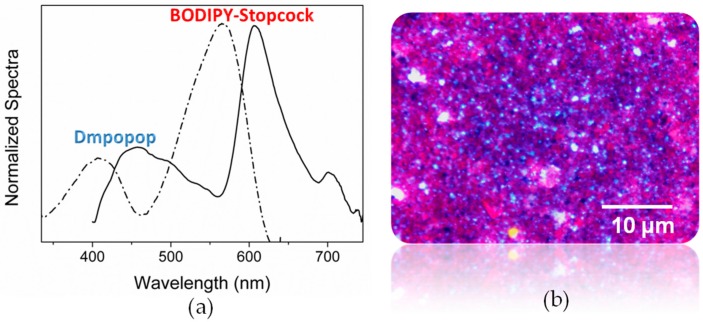
(**a**) Normalized excitation (emission measured at 660 nm, dashed line) and emission (excited at 350 nm, solid line) spectra of DMPOPOP enclose into the channels and BODIPY stopcock grafted in the disc-shaped LTL zeolite entrances; (**b**) Fluorescence microscopy image of the hybrid zeolite-dye system under 350/50-nm band-pass excitation and recording the emission with a 400-nm long-pass filter.

Once having checked that the designed closure molecule is valid, we proceeded to build the antenna system by its combination with a suitable energy donor. In this sense, Dmpopop has been again chosen because it has a broad absorption band, and its emission band overlaps well with the absorption band of the stopcock (see Figure S3 in the Supporting Information) so as to undergo FRET (Table 1). Thus, the Dmpopop donor has been allocated inside the channels, and afterwards, the entrances were plugged with the silylated BODIPY. To this aim, disc-shaped LTL zeolite has been again selected owing to its morphology, characterized by a basal surface much higher than the coat (low aspect ratio, <1; see Section 3.2 for details), which ensures that the stopcock molecules tend to react mainly with the silanol groups of the channel entrances rather than those on the coat. Selective excitation of Dmpopop leads to a predominant red fluorescence from the stopcock at around 610 nm (Figure 8), supporting the ongoing FRET process from the inside to the outside of the crystal along the channel direction. Further evidence is gathered by the corresponding excitation spectrum monitored at long wavelengths, where just the stopcock emits. Indeed, two clear bands are recorded, that expected from the silylated BODIPY at lower energies and another at higher energies from the occluded Dmpopop donor. It is noteworthy that the FRET is still highly efficient, albeit the donor-acceptor distance should be longer than when both dyes are inside the channels. Likely energy migration between the donor molecules in the channels contributes to enlarging the effective distance for FRET and reaches the stopcock energy traps placed further away at the entrances.

Summing up, the coupling of an external stopcock fluorophore as the energy acceptor at the ends of the LTL zeolite channels allows a second stage of organization, in which the excitation energy is transported by donor molecules placed in the inner space of the channels and finally injected to the acceptor molecules placed at the entrances. Thus, the stopcock molecules enable the communication between the guest molecules allocated inside the pores with external materials or molecules outside and also prevent small molecules like H_2_O or O_2_ (fluorescence quenchers) from diffusing into the interior of the crystals.

## 3. Materials and Methods

### 3.1. Dyes

Laser-grade BODIPY dyes, (PM546 and PM567) were purchased by Exciton, while oxazine 1 and 4, oxazole (Dmpopop) and carbostyril (C165) were purchased by Across Organics and used as received without further purification. The BODIPY-based stopcock molecule presented in Section 2.2 was synthesized by Dr. Yi Xiao from Dalian University (Dalian, China). 

### 3.2. LTL Zeolite Synthesis

Zeolite crystals with a tunable size (from around 100 nm to 2 μm) and morphology (from barrels to discs or coins) were attained by adjusting the conditions of the hydrothermal synthesis assisted by microwave heating. Further details of the synthesis and characterization of the crystals can be found elsewhere [43]. In particular, in the herein reported work, disc-shaped zeolites (average thickness of 200 nm and average diameter around 1 μm) were used as hosts for the antennae, whereas barrels (length around 2 mm) were used for the measurements of stopcock localization under the fluorescence microscope.

### 3.3. Dye Incorporation

Previous to dye incorporation, the zeolite was pre-treated with cesium (Cs^+^) cations to decrease the characteristic acidity of the zeolitic channels and adjust the pH to avoid the degradation or parallel acid-base equilibria of the incorporated dyes. To this aim, the zeolite was suspended in water and exchanged with an excess of CsCl under reflux and stirring during 45 min at 80 °C. Cationic dyes (oxazine 1 and 4) were incorporated into Cs^+^-LTL zeolite by cationic exchange by means of the addition of an aqueous solution of the dye to a zeolite suspension in water at 50 °C and during 6 h under stirring and reflux. Neutral dyes (BODIPY, Dmpopop and C165) were incorporated into Cs^+^-exchanged LTL zeolite by using gas phase insertion at high temperatures. After vacuum drying of the zeolite, it was sealed together with the adequate quantity of the dye at vacuum in an ampoule. Then, the dye was sublimated in a rotary oven during 2 days at 170 °C and 200 °C for BODIPY and Dmpopop, and C165, respectively. As a last step, the dye-doped zeolite was rinsed with butanol several times to remove the molecules adsorbed onto the external surface of the crystal.

### 3.4. External Functionalization of LTL Zeolite

LTL zeolite crystals (100 mg) were suspended in 20 mL of toluene in a previously-silanized glass flask to render the surface inert and avoid any possible reactions between the stopcock molecule and the glass. The number of channel entrances in one crystal was calculated (twice the number of pores, which are around 267,000 for the herein tested disc-shaped crystals of 1-µm diameter) [32], and one equivalent of BODIPY-stopcock molecule was added to the suspension. In the next step, the solution was sonicated for 15 min to enable the adsorption of the stopcock on the LTL zeolite surface and then heated at reflux for 15 h (Figure 6). Finally, the crystals were centrifuged and washed twice with methanol to remove the excess of stopcock molecules adsorbed on the surface.

### 3.5. Dye Loading Level 

The amount of dye incorporated in the LTL zeolite channels for a certain loading was estimated using the theoretically-calculated (semiempirical AM1) molecular dimensions of the molecule and the unit cell parameters provided by X-ray diffraction [32]. Considering the length of the long axis of the molecule, the adsorption site filled for each dye can be estimated (i.e., the number of unit cells occupied by a dye molecule), being two unit cells for carbostyril, BODIPY and oxazine, whereas three for oxazole. Thus, knowing the number of unit cells in the zeolite sample, the target dye loading is calculated according to the number of adsorption sites desired to be occupied. 

### 3.6. Spectroscopic and Microscopic Characterization 

The absorption spectra in the solid state (powder) were registered in a Varian spectrophotometer (Model Cary 4E, Agilent Technologies, Santa Clara, CA, USA) by an integrating sphere in which the reflectance is detected instead of the transmittance. The fluorescence steady-state spectra were recorded in the solid state using the front-face configuration in an spectrofluorometer (Model FLSP 920, Edinburgh Instruments, Livingston, UK) and corrected from the monochromators’ wavelength dependence, the lamp profile and the photomultiplier sensitivity. Fluorescence images of dye-doped zeolites were recorded with an optical inverted microscope with epi configuration (Olympus BX51) equipped with a color CCD (DP72). Samples excitation was set by a Chroma band-pass filter (350/50), and emission was collected with a Chroma cutoff filter (E400LPv2 from 400 nm). The corresponding fluorescence spectra were recorded by a fiber coupling from Olympus microscope to the aforementioned spectrofluorometer.

## 4. Conclusions

The strategy of allocating suitable dyes into the pores of LTL zeolite or plugging the entrances of LTL zeolite with fluorophores has been proven as a successful approach to achieve highly organized bioinspired antenna materials guided by FRET processes, in which dyes are protected and aligned in a preferential disposition avoiding fluorescent-quencher aggregates even at high loadings. Such a cage-protecting effect leads to a highly photostable and reliable material. Indeed, no sign of degradation was detected, neither after long irradiation periods (even with strong light sources as diode lasers), nor with the aging-time (up to several months). These photoactive hybrid materials are able to harvest light from a wide range of the electromagnetic spectrum (UV-Vis) and convert it into red light. Moreover, the overall outcoming fluorescence from the hybrid material can be triggered by just choosing the right dye combination and amount. Parameters such as the donor/acceptor spectral overlap and ratio, ruling the FRET efficiency, and the brightness of the final emitting acceptor, play a key role in this regard. A fine control of such features gives rise to very versatile photoactive materials where not only predominant red emission can be obtained, but also white-light emission (simultaneous blue, green and red emission). Accordingly, the combination of ameliorated FRET efficiency and acceptors with improved fluorescence efficiencies is required to push the antenna effect and consequently achieve dominating red emission, whereas lowering the acceptor content (to decrease the FRET efficiency for a given dye mixture) or choosing a final energy trap with lower emission ability is mandatory to attain white-light-emitting materials, where the light can be modulated over a broad spectral range.

The functionalization of the channels entrances with closure molecules based on BODIPY dyes endows further versatility to the material. Indeed, the herein reported stopcock has free chromophoric positions amenable to post-functionalization, rendering multifunctional and smart nanomaterials. As a matter of fact, the methyls groups at the α-pyrrolic positions can undergo Knoevenagel condensations, which allow the attachment of aromatic frameworks to the dipyrrin backbone and push the spectral bands deeper into the red part of the visible spectrum or eventually reaching the NIR region. Alternatively, the fluorine atoms of the boron bridge can be replaced through nucleophilic substitutions. In this way, suitable functionalized chains can be grafted at this boron center, without altering the photophysics of the dye, to promote the coupling of the dye-doped material with the target device (i.e., semiconductor for photovoltaics purposes) or to link zeolites to each other. Current work is in progress to test these avenues.

## Figures and Tables

**Table 1:** Meaningful data about dye loadings, spectral band positions and the ongoing FRET between the dyes occluded or anchored into the disc-shaped LTL zeolite: dye loadings, donor-acceptor ratio, spectral overlap (J), absorption (λ_ab_) and fluorescence (λ_fl_) peak wavelengths. C165, carbostyril; Ox, oxazine.

	Dyes	Dye Loading ^a^ (%)	Dye Ratio	J ^b^ (10^−13^ M^−1^ cm^3^)	R_0_^c^ (Å)	λ_ab_ (nm)	λ_fl_ (nm)
**D1:D2:A**	Dmpopop:PM567: Ox1	10:10:10	1:1:1	D1-D2 = 1.0	55.0	364	429
				D2-A = 1.8	65.8	518	539
						646	669
	C165:PM546: Ox4	10:10:10	1:1:1	D1-D2 = 1.1	59.1	360	424
				D2-A = 2.0	67.0	495	509
						614	638
		10:10:2	1:1:0.2	D1-D2 = 1.1	59.1	360	424
				D2-A = 2.0	67.0	495	509
						614	638
**D:A**	Dmpopop: Stopcock	10:*	1:0.05	0.06	34.4	364	436
						558	599

^a^ The dye loading (%) is calculated on the basis of the occupied adsorption sites by the encapsulated dyes (see Section 3.5). * The amount of stopcock grafted to zeolite was calculated on the basis of the available pore entrances (calculated as twice the number of channels; see Section 3.4). ^b^ The spectral overlap between the donor fluorescence spectra and acceptor main absorption band for each pair was calculated according to the Förster formalism [13]. ^c^ Critical transfer distance at which the FRET efficiency is 50%, calculated according to the Förster formalism assuming an orientational factor κ^2^ of 1 [13].

## Supplementary Materials

The following are available online at www.mdpi.com/1996-1944/10/5/495/s1: Figure S1 to S3: Spectral overlaps between energy donor and acceptor enabling FRET in each tested dye combination.
