# Supplementary material for: Photoactive Nanomaterials Inspired by Nature: LTL Zeolite Doped with Laser Dyes as Artificial Light Harvesting Systems
Source: Materials (Basel). 2017 May 4;10(5):495. doi: 10.3390/ma10050495 (PMC5459014; doi:10.3390/ma10050495)
Supplement: Supplementary file 1 [file materials-10-00495-s001.pdf]

Supplementary Materials: Photoactive Nanomaterials inspired by Nature: LTL zeolite doped with laser dyes as artificial light harvesting systems

Leire Gartzia-Rivero, Jorge Bañuelos and Iñigo López-Arbeloa

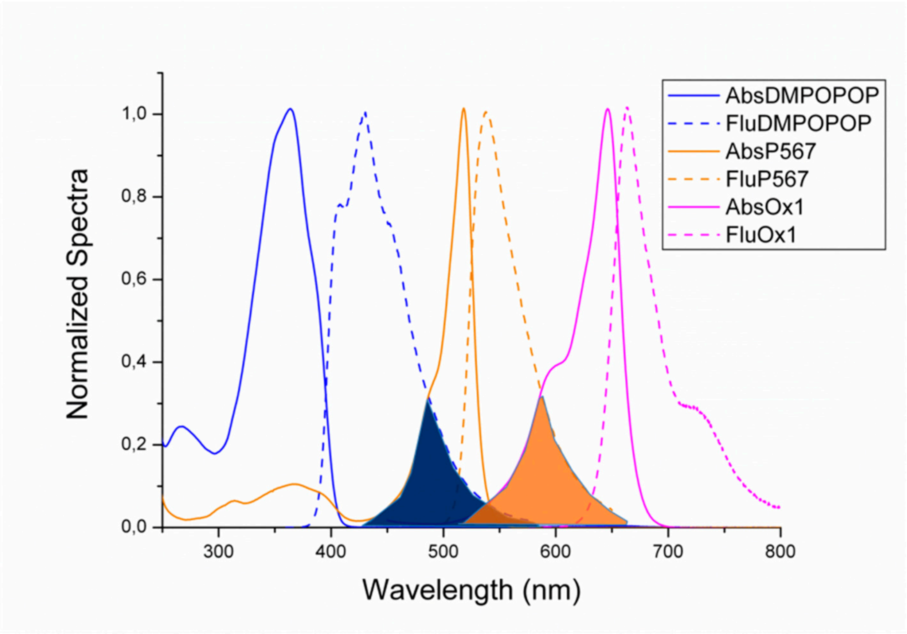

Figure S1. Normalized absorption and fluorescence spectra of the fluorophores Dmpopop, PM567 and Oxazine 1 in solution showing the corresponding spectral overlaps.

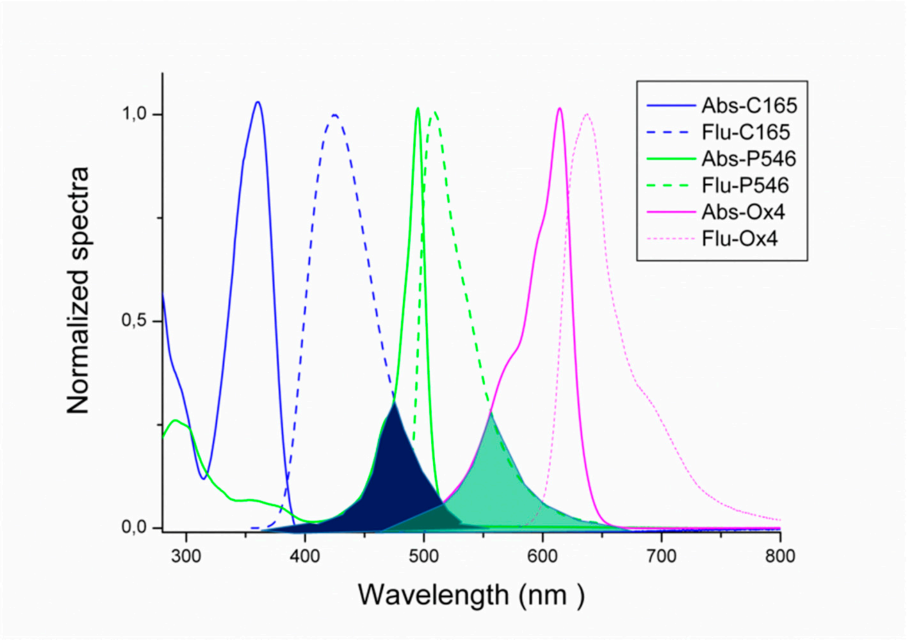

Figure S2. Normalized absorption and fluorescence spectra of the fluorophores C165, PM546 and Oxazine 4 in solution showing the corresponding spectral overlaps.

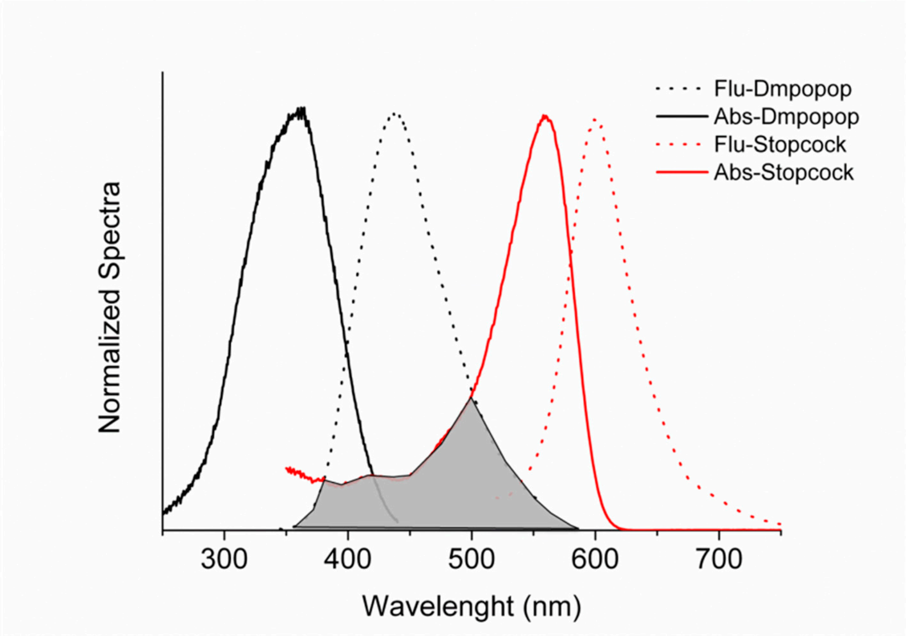

Figure S3. Normalized absorption and fluorescence spectra of the fluorophores Dmpopop and BODIPY-stopcock showing the corresponding spectral overlap.
